# Timbre perception in violins: spectral and formant influences of string materials in a controlled study

**DOI:** 10.1038/s41598-025-23548-0

**Published:** 2025-11-13

**Authors:** Takumi Asakura

**Affiliations:** Department of Mechanical and Aerospace Engineering, Faculty of Science and Engineering, Tokyo University of Science, Chiba, Japan

**Keywords:** Violin timbre, String materials, Subjective evaluation, Acoustic analysis, Machine learning, Formant frequencies, Spectral energy, Gradient boosting tree, Psychology, Mechanical engineering

## Abstract

String instrument timbre is influenced by a complex interplay of string material, instrument body characteristics, and playing technique. However, the perceptual effects of different string materials and their relationship with acoustic parameters remain incompletely understood. This study investigates how violin string materials (gut, nylon, and steel) affect subjective timbre perception and their correlation with acoustic features. Violin sounds were recorded using an automated bowing machine, ensuring consistency, and analyzed for spectral energy distribution and formant frequencies. Subjective impressions were collected using a semantic differential (SD) method, and factor analysis identified three key perceptual dimensions: Texture, Sharpness, and Power. Statistical analysis revealed that string material significantly influenced perceived timbre, with gut and nylon strings associated with darker and rougher tones and steel strings producing brighter and more defined sounds. Correlation analysis showed that Timbre perception was negatively associated with formant frequencies, while Sharpness was influenced by high-frequency energy levels, particularly in the mid-to-high range. Power exhibited weaker correlations with acoustic parameters, suggesting additional influences from temporal dynamics and instrument-specific characteristics. A Gradient Boosting Tree (GBT) regression model was employed to predict subjective impressions from acoustic parameters, demonstrating superior performance over linear regression methods (*R*² ≈ 0.47 for Timbre and Sharpness). Sensitivity analysis further confirmed that formant frequencies and spectral balance played a critical role in shaping timbre perception. These findings provide valuable insights into the perceptual mechanisms of violin timbre, benefiting musicians, instrument makers, and computational timbre modeling research.

## Introduction

String instrument sound is shaped by the interplay of string vibrations, instrument body resonance, and playing technique^1,2^. Nonlinear string vibration significantly affects spectral qualities, particularly in bowed instruments like violins, where bowing technique modulates tone richness^3,4^. Body resonance further filters overtone frequencies, enhancing tonal quality^5^.

Instrument-string interactions crucially integrate structural elements and performance techniques. Bowed strings alternate between stick and slip modes, generating periodic oscillations^6^. Structural components like soundboards and resonating chambers further influence tone^2,7,8^. Performers rely heavily on tactile feedback, influenced by string diameter and tension^9,10^.

Acoustic properties and materials strongly influence sound quality. For example, in studies on cello strings, string stiffness has been shown to enhance high frequencies and alter timbre above 600 Hz^11^. Although differences in resonance structure exist between cellos and violins, such findings offer useful insight into how material properties may influence timbre in bowed instruments more generally. Instrument materials such as wood also critically shape timbre; density, Young’s modulus, and internal friction determine resonance and tonal clarity^12–15^. String material significantly impacts acoustic and mechanical properties, historically exemplified by gut strings favored for tonal complexity despite environmental instability^16–18^. Steel strings gained popularity post-WWI due to durability and tuning stability^16,19,20^. Nylon strings, introduced later, offer stable tuning, softer tone, and easier playability, notably in classical guitars^21,22^. Advancements in string design include optimizing tension and density, addressing inharmonicity through non-uniform strings, and employing synthetic materials and digital modeling for richer tonal control^23–26^.

Understanding string properties in relation to timbre perception is essential. Acoustic descriptors such as spectral balance and sharpness explain timbral differences^27,28^. Machine learning approaches enhance timbral modeling using perceptual features and acoustic descriptors^27,29^, benefiting music education and evaluation^31^.

However, gaps remain regarding how listeners perceive different string materials. Existing research focuses largely on physical properties without directly linking them to perceptual evaluations. To address this limitation, this study investigates: (1) the impact of string materials on perceived timbre, and (2) correlations between acoustic parameters and human perception. Combining acoustic analyses with subjective evaluations, the results will provide comprehensive insights beneficial to musicians, luthiers, and acoustics researchers. Notably, Saitis et al.^32^ conducted a psycholinguistic analysis of musicians’ verbal descriptions during violin preference evaluations. Although their approach differs from our method based on the semantic differential (SD) scheme, their findings support the relevance of semantic constructs in musical timbre evaluation contexts.

## Materials and methods

### Research flow

This study recorded violin sounds with an automated bowing machine for consistency, followed by spectral and formant analyses. Subjective evaluations using the SD method identified key perceptual dimensions via factor analysis. Gradient Boosting Tree (GBT) regression modeled nonlinear relationships between acoustic features and impressions, with a final sensitivity analysis assessing acoustic parameter influence.

## Instruments and strings used in the experiment

This study examined the characteristics and tonal qualities of 13 different D strings, comprising four gut strings, four nylon strings, and five steel strings, as listed in Table 1. Additionally, it investigated the influence of different instruments on tonal variation. The string types were categorized as gut (GT), nylon (NL), and steel (ST), each assigned a sequential identifier.

**Fig. 1:**
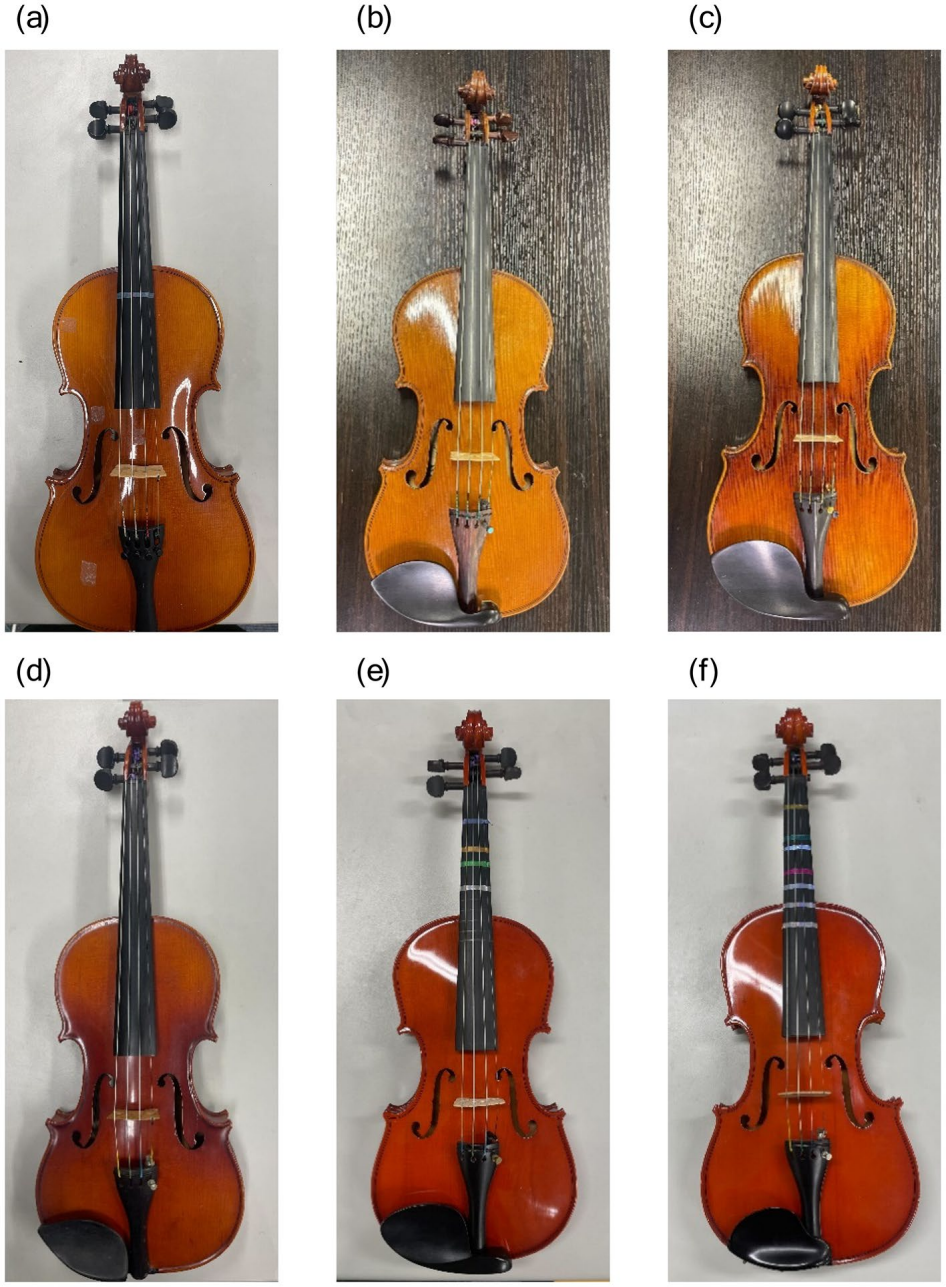
Violins used in the experiment. Each violin (**a**-**f**) corresponds to instruments A-F, as detailed in Table 2.

To assess the impact of instrument variation, six violins differing in manufacturing period and country of origin, as detailed in Table 2; Fig. 1, were used for recording. This allowed for a comparative analysis of the tonal differences introduced by each instrument.

**Table 1:**
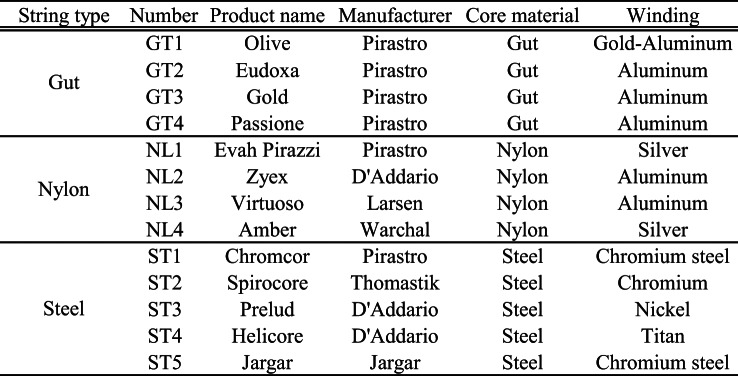
Specifications and classification of strings used in the experiment, including material and type.

**Table 2:**
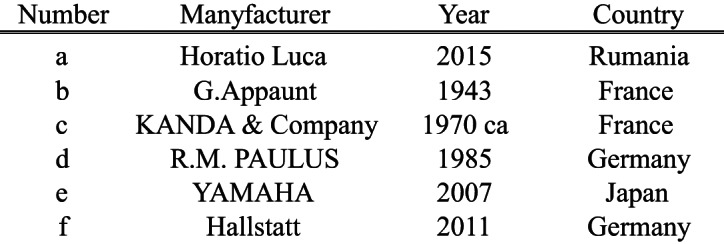
Specifications of Violins Used in the Experiment, Including Manufacturer, Year of Production, and Country of Origin.

## Acoustic measurement

**Fig. 2:**
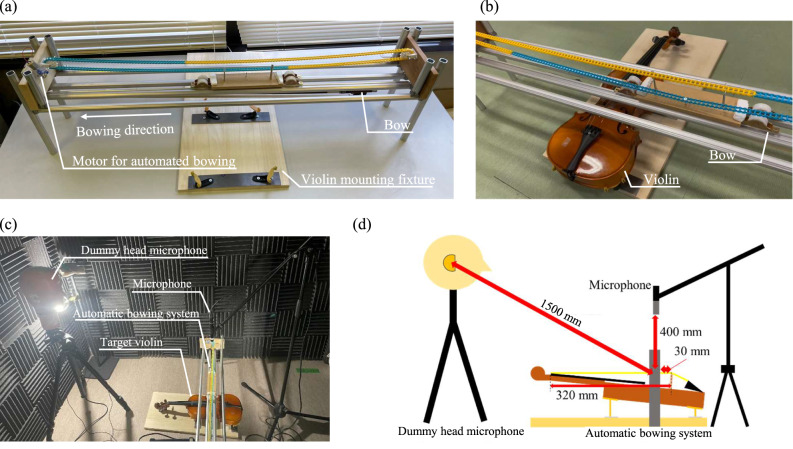
Experimental setup for violin sound analysis using an automated bowing system. (**a** and **b**) Close-up views of the automated bowing mechanism, including the motorized bowing system, violin mounting fixture, and bowing direction. (**c**) Overview of the recording setup, featuring the automated bowing system, target violin, and microphone arrangement, including a dummy head microphone for binaural recordings. (**d**) Schematic representation of the experimental setup, illustrating the spatial arrangement of the automatic bowing system and microphones.

This study experimentally examined the frequency spectra produced when different strings and instruments were played. The sound production mechanism of the violin involves bowing the strings attached to the body, causing them to vibrate. To eliminate variations in bowing speed and pressure, an automated bowing device was developed, as shown in Fig. 2(a). The bowing motion was mechanically controlled, as depicted in Fig. 2(b), ensuring consistent bowing speed and position across all strings. We deliberately fixed bowing speed and normal force to reduce nuisance variability and isolate string- and instrument-dependent spectral structure. This setting approximates a controlled *mf*–*mp* condition and improves comparability across 78 string–violin combinations. We acknowledge that using a single speed/pressure limits ecological variability; the Discussion explicitly qualifies generalization accordingly.

For sound recording, two different setups were used: a unidirectional microphone (ECM-100U, Sony Corporation, Tokyo, Japan) for acoustic analysis and a dummy head (Type 8328B, ACO CO., LTD., Chiba, Japan) for subjective evaluation experiments. The recordings were captured using an audio interface (UR44C, Steinberg Media Technologies GmbH, Hamburg, Germany) and analyzed on a computer. The Sony ECM-100U microphone, being uni-directional and based on a pressure-gradient transducer design, may exhibit a proximity effect that emphasizes low-frequency components depending on distance. To minimize such influence, the microphone was placed at a fixed distance of 400 mm from the bowing point. We acknowledge that some low-frequency emphasis may have occurred and have added this as a limitation. The placement of the measurement equipment is shown in Figs. 2(c) and 2(d). The measurements were conducted in a semi-treated recording room with sound-absorbing wall panels and an untreated floor. The room dimensions were approximately 4.5 m (width) × 5.0 m (depth) × 3.0 m (height), and the reverberation time (T60) was measured to be approximately 0.3 s. While not a fully anechoic chamber, the environment was sufficiently controlled to minimize early reflections and standing waves in the mid-frequency range.

The microphone was positioned 400 mm from the bowing location to record the sounds produced by each string under the automated bowing conditions. Each string–instrument combination was recorded once using the automated bowing apparatus under standardized conditions. Because the bowing mechanism was mechanically controlled with fixed pressure and position, and the tuning was verified with high-precision digital tuners prior to each recording, the resulting audio samples were highly consistent. The system ensured minimal variation across recordings, providing stable input for both acoustic and perceptual analyses. The total string length was 320 mm, and the bowing position was set such that the bow’s center was 30 mm away from the bridge. This 320 mm vibrating string length was standardized across all violin and string combinations by adjusting each instrument’s bridge position using precision digital calipers, ensuring consistent scale length. To minimize tuning variability, the third string (D4) was tuned to 293.7 Hz using a chromatic digital tuner with ± 0.5 cent resolution. Tuning was checked and adjusted immediately before each recording session to maintain pitch accuracy throughout data collection. Note that the bowing speed was approximately 80 mm per second. The bow was held in contact with the string using a fixed mechanism designed to apply a constant downward force, ensuring uniform bowing pressure across all trials. The bowing parameters adopted in this study were chosen to represent a moderate dynamic condition (*mf*–*mp*). These values fall within the typical ranges observed in previous studies. Askenfelt^33^ reported that, under normal playing conditions, bow velocities typically ranged from 0.1 to 1 m/s—with the lowest stable tone produced at approximately 0.04 m/s. Later performance analyses^34^ also described comparable regions for mid-dynamic bowing, although they did not specify a particular average value. Accordingly, the present settings were selected to lie well within the empirically observed playable range for moderate dynamics.

For binaural recording, the dummy head was positioned 1.5 m from the violin bridge at ear height, facing forward (0° elevation angle on the median plane). The violin was located approximately 45° below the horizontal line of sight of the dummy head, resulting in a downward incidence angle. This geometry may have influenced the recorded spectral characteristics due to direction-dependent head-related transfer functions (HRTFs). The sound of D4 (293.7 Hz) was recorded as an open string, then tuning was verified before each take. The sampling frequency was set to 48 kHz, and the FFT size was 2^{15} (32,768). The frequency spectra were analyzed to compare the characteristics of different strings and instruments. The signals were recorded at 24-bit resolution to ensure high dynamic range and low quantization noise. For spectral analysis, we employed short-time Fourier transform (STFT) representations to compute band power and derive formant-like features. Power spectral density (PSD) estimates were not used.

The same sets of strings (gut, nylon, and steel) were used across all six violins to ensure comparability. Strings were installed sequentially from Violin 1 through Violin 6 for each material type. To minimize wear-related variation, each string was carefully inspected before and after each use, and replaced if any signs of fatigue or deformation were observed. Additionally, tuning and tension were re-checked for each recording session. While full counter-balancing of installation order was not feasible due to logistical constraints, every effort was made to standardize handling and minimize cumulative effects.

## Psychological measurement

**Fig. 3:**
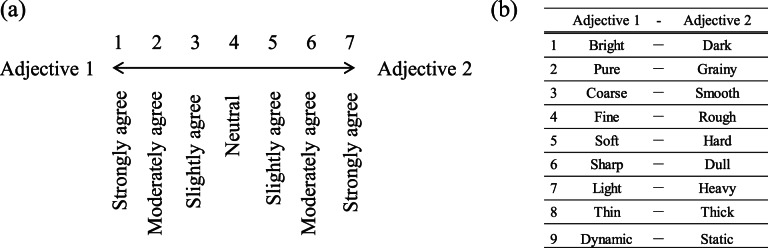
(**a**) The seven-point Likert scale used in the SD experiment and (**b**) the adjective pairs representing the two poles, Adjective 1 and Adjective 2, respectively.

The subjective impressions of the simulated acoustic stimuli were measured by a subjective evaluation test using the SD method^35^. Participants listened to the stimuli via high-fidelity Audio-technica ATH-WP900 headphones, dichotically driven by the built-in headphone amplifier of a Focusrite Scarlett 2i2 USB audio interface. In this evaluation, a seven-point Likert scale was used (Fig. 3(a)). Then, the 9 adjectives shown in Fig. 3(b) were used. The Thin vs. Thick scale was referenced from^36^, while Bright vs. Dark, Pure vs. Grainy, Coarse vs. Smooth, and Light vs. Heavy were adopted from^37^. The Soft vs. Hard, Sharp vs. Dull, and Dynamic vs. Static scales were taken from^27^. Additionally, since timbre is related to the texture of physical surfaces^38^, Fine vs. Rough was referenced from^39^.

In the subjective impression evaluation experiment, the recorded sound data was used. Participants were instructed to evaluate the stable portion of each sound clip based on its perceptual attributes (i.e., timbre-related impressions), such as brightness, roughness, and clarity.

Each participant rated 78 stimuli (13 D strings × 6 violins; one open-string D4 recording per string–violin combination) in a single session. Stimuli were presented once each in randomized order with short self-paced breaks every ~ 10–15 trials. Participants could request one replay per trial. Each trial required 9 SD ratings (7-point scales). The total session length per participant was approximately 40 min. Thus, per participant we obtained 78 ratings per adjective and 78 factor scores after factor-based aggregation. To mitigate fatigue and order effects, the presentation order was newly randomized for each participant.

Each stimulus was rated once per participant; within-participant variability was therefore handled analytically (via factor aggregation and cross-validated prediction) rather than by repeated presentations. Session length and scheduled breaks were chosen to minimize fatigue while maintaining attention.

## Acoustic analysis

The generated sounds were analyzed using acoustic energy across different frequency bands and formant frequencies as acoustic parameters. These parameters served as observed variables to explain the subjective impressions of the sounds. The frequency bands were selected based on previous research^29^, which has been used for violin acoustic evaluation. The specific frequency bands analyzed were 190–380 Hz (Band-1), 380–760 Hz (Band-2), 760–1520 Hz (Band-3), 1520–3040 Hz (Band-4), and 3040–6080 Hz (Band-5), with corresponding center frequencies of 285, 570, 1140, 2280, and 4560 Hz, respectively. These bands are hereafter referred to by their center frequencies.

The Praat software (version 6.2) was used to extract spectral envelope peaks from the violin sound waves. Formant frequencies were obtained from the stable portions of the recorded waveforms, capturing the first (F1) to fourth (F4) formants. These formants are hereafter referred to by their typical frequencies of F1 (500 Hz), F2 (1000 Hz), F3 (2000 Hz) and F4 (3000 Hz), respectively. The initial attack and decay portions of the waveform were excluded from the analysis, focusing only on segments with stable amplitude characteristics for acoustic evaluation. Although ‘formant’ typically refers to vocal tract resonances, in this study, we use it to describe spectral envelope peaks that correspond to resonant modes of the violin body or air cavity. This interpretation aligns with prior research mapping spectral structure to timbral impressions in musical instruments.

In this study, “F1–F4” denote peaks of the spectral envelope detected by Praat’s *Formant (burg)* algorithm; they are not intended to represent vocal-tract formants^40,41^. For each sustained tone we analyzed the steady portion (300–1300 ms) and extracted F1–F4 with: maximum number of formants = 4, maximum formant = 5000 Hz, window length = 0.05 s, time step = 0.01 s, pre-emphasis from = 50 Hz.

Violins also exhibit low-frequency body/air resonances—the air-cavity A0 ≈ 280–300 Hz and several body/plate modes around 400–800 Hz—which may fall outside the nominal F1 track under LPC settings tailored to sustained tones^42,43^. We therefore quantify these low-frequency regions via band-energy descriptors, namely Band-1: 190–380 Hz (covering A0) and Band-2: 380–760 Hz (covering body/plate modes). Accordingly, F1–F4 are interpreted as concise mid-band envelope summaries, not literal “violin formants.”

### Participants

Twenty Japanese individuals (10 males and 10 females; mean age: 24.9 ± 6.6 years) participated in the experiment. To confirm the normal auditory and visual abilities of participants in the experiment, a self-reported questionnaire survey was conducted in advance. Participants who answered without any hearing problems were selected. Additionally, to ensure a more accurate hearing assessment during the experiment, the WHO-provided hearing test app hearWHO was used. Participants who recorded a score of 50 or higher were considered to have normal hearing and were included as subjects in the experiment.

The experimental protocol adhered to the “Ethical Principles Based on the Declaration of Helsinki” and the “Ethical Guidelines for Medical Research Involving Human Subjects” (Ministry of Health, Labour and Welfare of Japan). Furthermore, the study was conducted in compliance with the International Standard Audiovisual Number EN 50332-1 and − 2, proposed by the European Committee for Electrotechnical Standardization^44,45^, as sound pressure regulations for portable audio players. This ensured the study was noninvasive. Informed consent was obtained from all participants after they were briefed on the purpose of the study and the experimental methods. Participants were also informed about the anonymization and use of their data.

## Factor analysis

To identify the factors concerning the subjective impressions of the presented binaural auditory environments, a factor analysis^46^ with Promax rotation was performed using JMP, Version 16 (SAS Institute Inc., Cary, NC, USA). The number of factors was determined comprehensively based on both the Kaiser–Guttman criterion^47^ and the elbow method.

After the factor analysis, a score for each factor was calculated for every participant by averaging their ratings across the corresponding adjectives. This provided a single factor score per acoustic stimulus per participant, allowing for an efficient and integrated evaluation of how each violin sound influenced the timbre impressions.

## Model selection and regression analysis

To model the relationship between the acoustic characteristics of violin sounds and subjective evaluation metrics, multiple regression techniques were evaluated using the screening function in JMP. The input variables were the acoustic features described in the previous sections, while the output variables consisted of SD scores and factor scores. The regression methods evaluated are listed in Table 3.

To identify the most suitable method, model selection was based on the coefficient of determination (*R*²) and Root Average Squared Error (RASE). A lower RASE indicates smaller prediction errors and is used as a comprehensive metric for model performance evaluation^48^. As a result, GBT was selected as the most predictive and best-fitting model. In addition, we report 10-fold cross-validation (stratified by string material) using the same GBT hyperparameters as in the main analysis. To assess robustness beyond chance, we ran a permutation test: the target factor scores were randomly shuffled 100 times while keeping predictors and a fixed 90/10 validation split unchanged. For each permutation we computed the cross-validated R²; the empirical p-value was calculated as the proportion of permuted R² values exceeding the observed R².

To characterize the response surface of the selected GBT in the psychological evaluations, we implemented a stochastic sensitivity analysis in JMP (Simulation Experiment). For each focal predictor (each band-level amplitude and each formant), we generated pseudo-random draws within empirically observed bounds (Simulation Count = 1000; Factor Space Proportion = 0.5), while holding all other predictors at string-type–specific medians. Sampling was restricted to the empirical data envelope to avoid extrapolation. Categorical factors were not treated as continuous; when string type was fixed, instrument identity was marginalized by scoring the model at all six instrument levels for each sampled input and taking an equal-weight mean, yielding an expected response for a typical instrument of that string type. Predicted factor scores (standardized to unit variance) were computed for all samples and summarized as sampled prediction maps showing how the model output changes over a natural dynamic range. Note that these displays are model-based predictions, not interpolations of single-shot ratings; the apparent continuity arises because we score the trained GBT at many pseudo-random inputs within the empirical range. Interpretation: greater local change across nearby sampled inputs indicates higher sensitivity, whereas near-uniform regions indicate lower sensitivity.

**Table 3:**
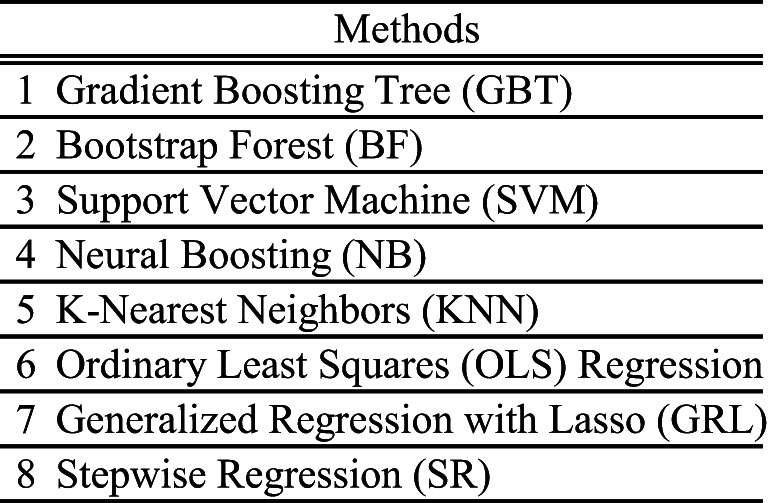
Regression methods evaluated for modeling the relationship between acoustic features and subjective impressions.

### Statistical analysis

All statistical analyses in this study were performed using JMP. First, to quantitatively evaluate the influence of physical acoustic parameters on subjective parameters, a linear-mixed-model-based (LMM-based) analysis of variance (ANOVA) was conducted. The main effect of the kind of instrument and string and the interaction between them were considered. In this analysis, subjective parameters were set as dependent variables, while individual attributes was set as fixed effects, and participant variability was considered a random effect to appropriately account for individual differences. Note that, prior to conducting ANOVA, the normality of each variable was assessed using the Shapiro–Wilk test. All variables satisfied the normality assumption and were therefore included in the analysis. Second, Spearman’s rank correlation coefficients were calculated for various pairs of subjective and objective parameters. Spearman’s rank correlation was employed to account for the ordinal nature of semantic differential ratings and to avoid assumptions of linearity inherent in Pearson correlation. These correlations were used to discuss interrelationships among the psychological and physical factors. For all analyses, *p*-values < 0.05 were considered significant.

## Results

### Profile analysis

**Fig. 4:**
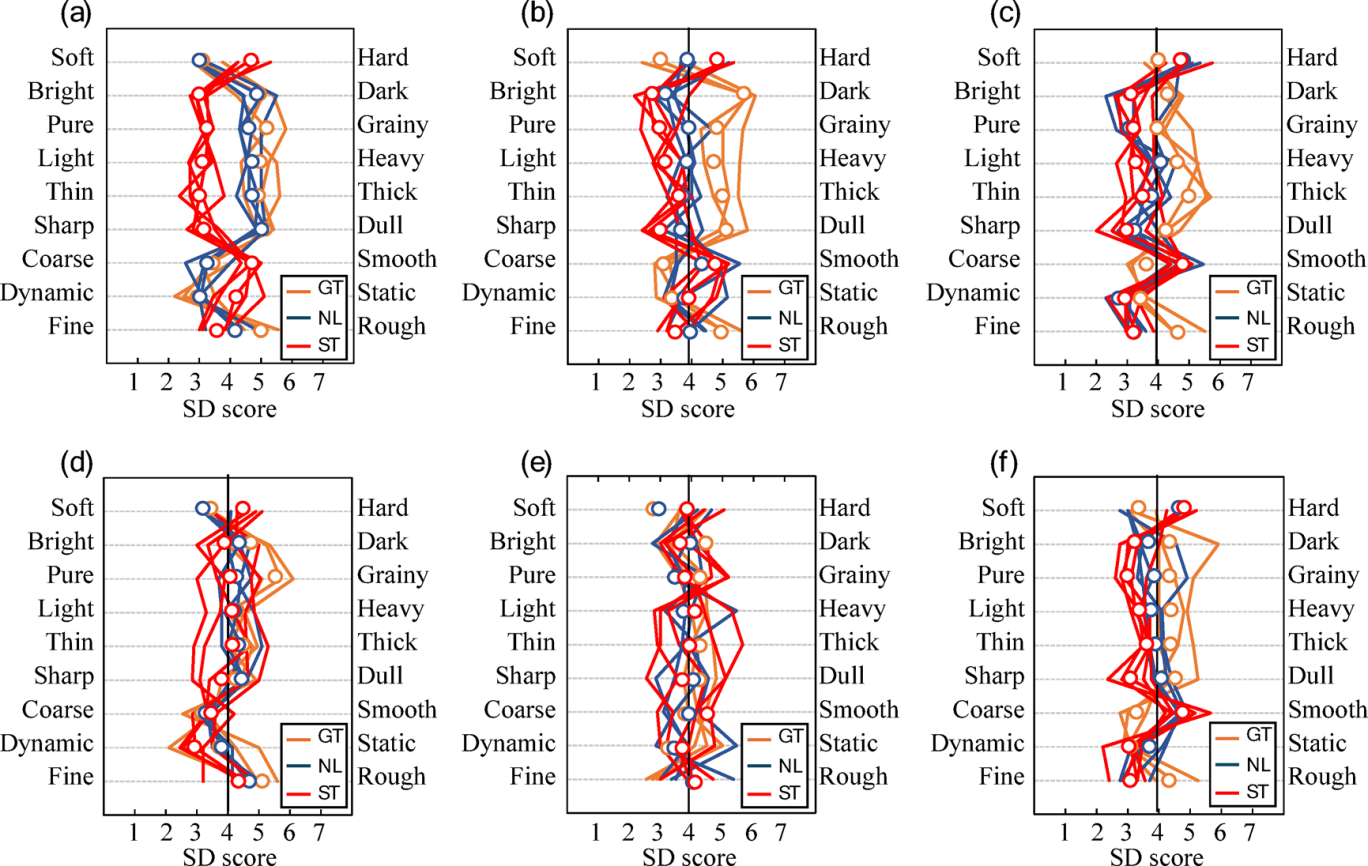
Mean semantic differential ratings across participants for each individual string exemplar. Results are grouped by instrument and string type. Open circles indicate means across string exemplars. Axes are aligned such that higher scores indicate greater perceptual intensity in the direction of the positive adjective (e.g., bright, sharp, powerful).

The SD scores for each instrument are shown in Fig. 4(a)–(f). The line graphs represent the average scores of all participants across all string types, while the open circles indicate the mean scores for each string type. Each color separates the kind of strings.

For Instrument A (Fig. 4(a)), comparing the evaluation results by string material reveals distinct trends. Gut strings received higher scores on scales such as Bright vs. Dark, Pure vs. Grainy, Light vs. Heavy, Thin vs. Thick, Sharp vs. Dull, and Fine vs. Rough, while all gut strings scored lower on Dynamic vs. Static. Nylon strings exhibited a similar trend to gut strings, showing high scores on Bright vs. Dark, Pure vs. Grainy, Light vs. Heavy, Thin vs. Thick, and Sharp vs. Dull, with mean values nearly identical to those of gut strings. In contrast, steel strings demonstrated a different tendency, scoring lower on Bright vs. Dark, Pure vs. Grainy, Light vs. Heavy, Thin vs. Thick, Sharp vs. Dull, and Fine vs. Rough, but higher on Coarse vs. Smooth and Dynamic vs. Static across all samples. These results suggest that Instrument A produces significantly different auditory impressions depending on whether gut/nylon strings or steel strings are used.

Examining the other instruments (Fig. 4(b)–(f)), Instrument B (Fig. 4(b)) exhibited a trend similar to Instrument A, with gut and steel strings showing symmetrical scores around the neutral point (score 4). However, unlike Instrument A, nylon strings in Instrument B displayed intermediate values between gut and steel strings. Instrument C (Fig. 4(c)) showed smaller differences in scores between steel and gut strings compared to Instruments A and B. The distinctions between steel and gut strings became even less pronounced in Instruments D and E, where the mean values for all three string types were nearly identical. Notably, the score variations for steel strings were particularly large. For Instrument F, the score trends for steel strings were relatively consistent across samples, whereas gut and nylon strings exhibited greater variability.

### Factor analysis

The results of the factor analysis are presented in Table 4. In this study, the number of factors was ultimately determined to be three based on the analysis results. The rationale for this decision is as follows.

**Fig. 5:**
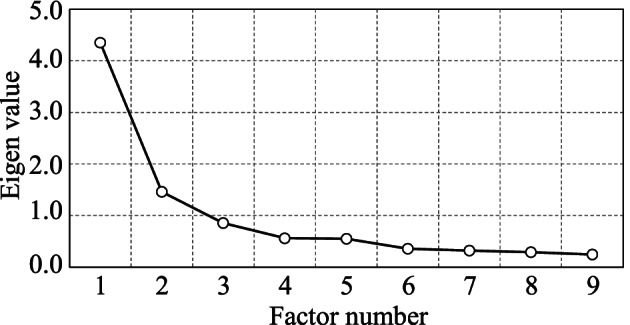
Scree plot showing the eigenvalues of the principal components extracted from the semantic differential ratings.

According to the Kaiser-Guttman criterion, only factors with an eigenvalue greater than 1 are typically retained. The eigenvalues obtained as Fig. 5 in this analysis were 4.35 for the first factor, 1.46 for the second, and 0.85 for the third, which would suggest retaining only the first two factors. However, the scree plot from the elbow method showed a clear inflection point at Factor 3, indicating the validity of including up to three factors.

Examining the cumulative contribution rate, Factor 1 alone accounted for 25.5% of the variance, Factors 1 and 2 together explained 43.7%, and including Factor 3 increased the explanatory power to 60.8%. Although previous studies have reported three to five perceptual dimensions for timbre using pairwise dissimilarity ratings^27,49^, the current study differs methodologically in that it employed direct adjective-based ratings of individual stimuli. These two approaches are not directly comparable, as they reflect different cognitive and perceptual strategies. Nevertheless, both methods aim to reveal the latent structure of timbre perception.

The factors and factor loadings for each adjective were obtained, with the highest scores among the three factors highlighted in red. Each adjective was then assigned to the factor for which it had the highest score. As a result, the three identified factors were associated with “Texture,” “Sharpness,” and “Power.” The selection of the label ‘Texture’ reflects a flexible approach to timbre semantics, in line with critiques of fixed models by Zacharakis et al.^50,51^. Hereafter, these three factors are referred to by their descriptive labels: *Texture* (Factor 1), *Sharpness* (Factor 2), and *Power* (Factor 3).

The Texture factor scores indicate that higher values correspond to darker, grainier, and coarser timbre. The Sharpness factor scores reflect harder and sharper tones, while the Power factor scores signify heavier, thicker, and weaker sounds.

**Fig. 6:**
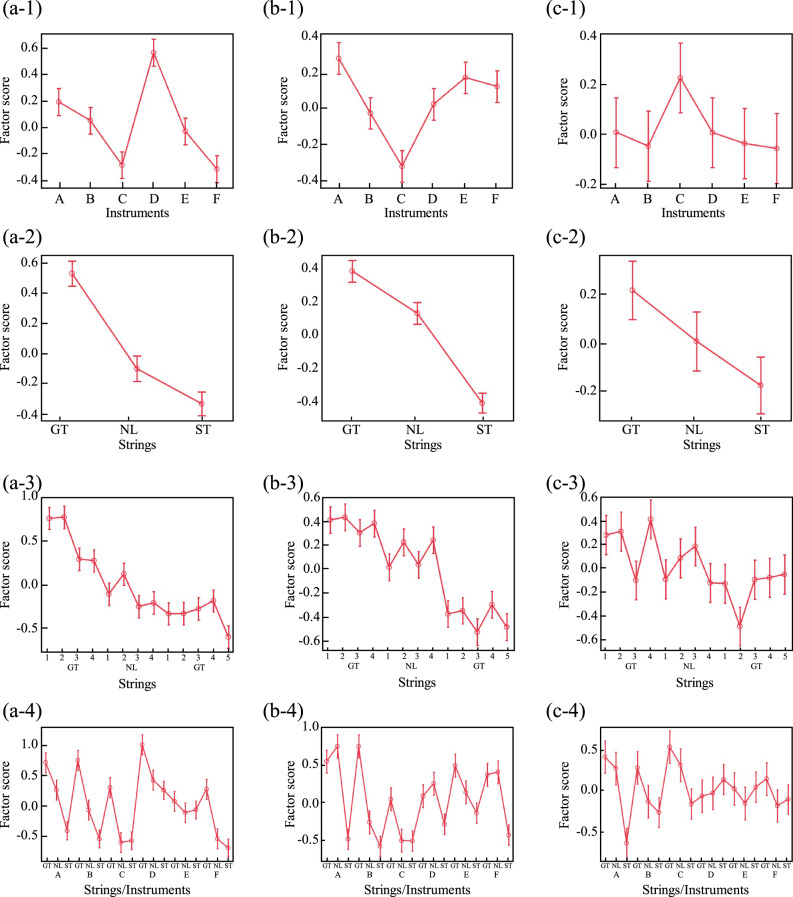
Relationship between the factor scores under each condition. (**a**), (**b**), and (**c**) represent three actors of Texture, Sharpness and Power, respectively. The numbers following hyphens after each letter indicate the specific experimental factor components.

To examine the effects of instrument type, string type, and their interaction on factor scores, an ANOVA was conducted, and the results are shown in Table 5. Significant main effects of both instrument and string type, as well as their interaction, were confirmed for all factors (*p* < 0.05). The corresponding means and standard errors are presented in Fig. 6.

**Table 4:**
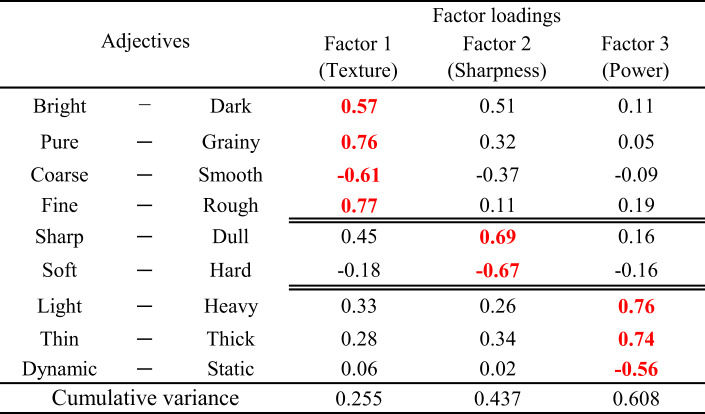
Factor loadings of adjectives for the three identified factors, where adjectives are grouped based on their highest loading among texture (Factor 1), sharpness (Factor 2), and power (Factor 3), with cumulative variance explained by each factor also presented.

**Table 5:**
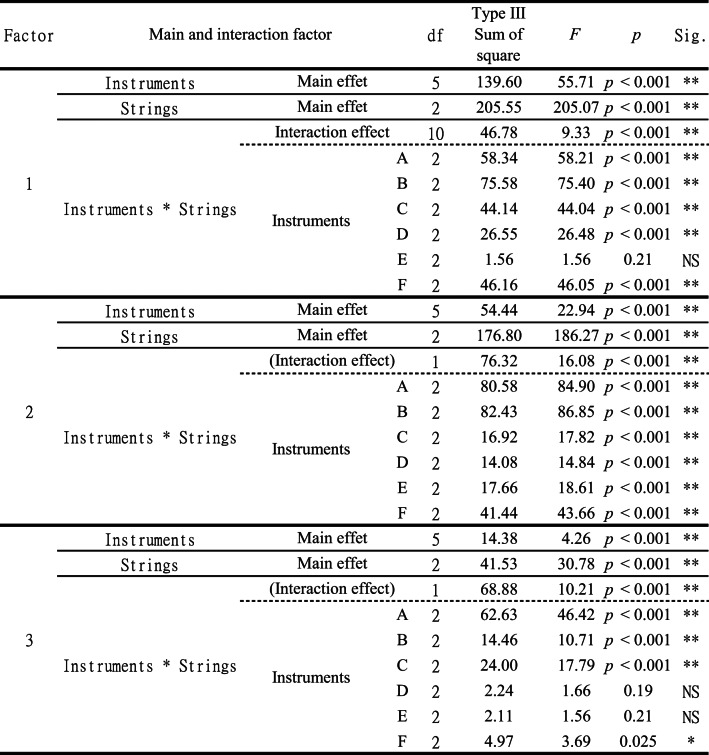
ANOVA results for instrument and string effects on perceptual factors, including the results of the Type III sum of squares ANOVA assessing the main effects of instruments and strings, as well as their interaction effects, on three perceptual factors. The degrees of freedom (df), sum of squares, F-values, and *p*-values are reported, with significance levels indicated as **: *p* < 0.001, *: *p* < 0.05, and non-significant results (NS).

Examining instrument-based differences in factor scores (Fig. 6(a-1)–6(c-1)), Instrument C and F showed lower Texture scores, whereas Instrument D had higher scores. Instrument C also exhibited notably lower Sharpness scores than the others, while its Power scores were higher. These differences suggest inherent tonal variations among instruments.

For string type differences (Fig. 6(a-2)–6(c-2)), factor scores generally decreased in the order of gut, nylon, and steel strings, with significant differences (*p* < 0.05) across all types. While nylon strings tended to have intermediate characteristics between gut and steel strings, some cases (b-2) showed nylon resembling gut strings more closely, while others (a-2) aligned more with steel.

Comparing factor scores across all strings (Fig. 6(a-3)–6(c-3)), steel strings showed low variation in Texture and Sharpness, suggesting that their characteristics remained consistent across different instruments. However, gut strings displayed greater variation, even within the same category. Power had the largest within-category variation, making inter-string differences less apparent. This suggests that, unlike Texture and Sharpness, Power-related characteristics are less consistently reproduced as acoustic features of strings.

Regarding instrument–string interactions (Fig. 6(a-4)–6(c-4)), while factor scores generally declined in the gut–nylon–steel order, deviations in ranking and conditions with minimal differences were observed depending on the factor and instrument. Simple main effect tests revealed that for Texture, the effect of string type was not significant (*p* > 0.05) in Instrument 5, and for Power, it was not significant in Instruments 4 and 5 (*p* > 0.05). This suggests that Instrument 5 may inherently suppress the characteristic differences between strings in terms of acoustic properties.

### Correlation between subjective and acoustic features

Table 6 presents the Spearman’s correlation coefficients between factor scores, acoustic energy in each frequency band, and formant frequencies, where positive correlations are shown in red, negative correlations in blue, and non-significant pairs (*p* > 0.05) as hyphens.

The correlations between factor scores are at most 0.23, indicating near-independence. For acoustic energy, Band-1 (285 Hz) shows a strong correlation (0.6–0.7) with other bands, while Bands 4 (2280 Hz) and 5 (4560 Hz) exhibit a moderate correlation (0.71), with other bands showing weaker correlations (0.2–0.3). Formant frequencies, however, display strong correlations (≥ 0.6) across all combinations, suggesting high interdependence.

Examining subjective–objective parameter correlations, Texture scores correlate negatively (~−0.3) with all formant frequencies, indicating that higher formant frequencies were associated with increased ratings on dimensions such as brightness and sharpness, which may be perceived as more expressive or vivid. However, these associations do not imply preference or desirability. In some psychophysical studies, correlations above 0.3 have been considered meaningful in specific contexts^52^. Although this does not universally imply statistical significance, it may still support the interpretability of our findings. Additionally, Band-5 (4560 Hz) shows a small negative correlation (~–0.2). Sharpness exhibits weaker negative correlations with formant frequencies and Bands 4 (2280 Hz) and 5 (456 Hz), while Power has few significant correlations.

**Table 6:**
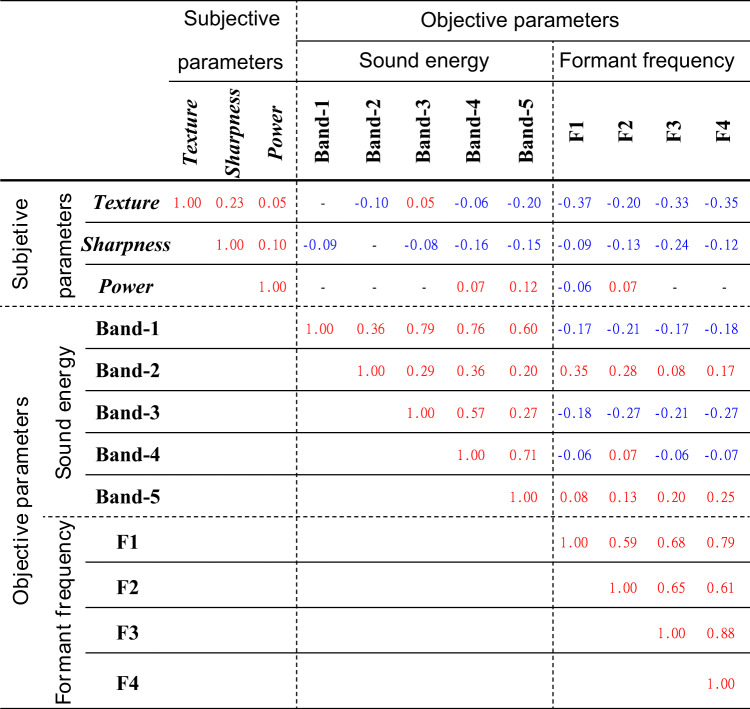
Spearman’s correlation coefficients between factor scores, acoustic energy across frequency bands, and formant frequencies, with positive correlations highlighted in red, negative correlations in blue, and non-significant correlations (*p* > 0.05) represented by hyphens.

### Modeling subjective impressions using acoustic features

The previous section confirmed weak to moderate correlations between subjective and acoustic parameters. However, correlation analysis only captures linear relationships, leaving potential nonlinear dependencies unexamined. To address this, regression performance was evaluated using the methods listed in Table 7.

The coefficient of determination (*R*²) for each regression model is shown in Table 7, ranked by *R*² (higher first) and RASE (lower first). The rankings remained consistent across conditions, with GBT achieving the highest performance, yielding *R*² ≈ 0.47 for Texture and Sharpness, while Power had slightly lower *R*².

GBT’s strong predictive power stems from its ensemble learning approach, where multiple decision trees iteratively correct errors, enhancing accuracy. Unlike linear regression or LASSO, GBT effectively captures nonlinear relationships, making it superior in modeling complex dependencies. Indeed, Stepwise regression produced *R*² as low as 0.1, while methods accommodating nonlinearity generally performed better, suggesting that subjective evaluations exhibit nonlinear dependence on acoustic parameters.

An *R*² of ~ 40% indicates that acoustic features explain ~ 40% of psychological variation, emphasizing their strong influence on subjective evaluation. However, the remaining 60% remains unexplained, likely influenced by individual factors such as musical experience, perception, cultural background, and measurement error.

In terms of explanatory power, Texture and Sharpness showed higher predictability, whereas Power had lower explanatory strength, indicating that Power is less directly represented by acoustic energy and formant frequencies.

**Table 7:**
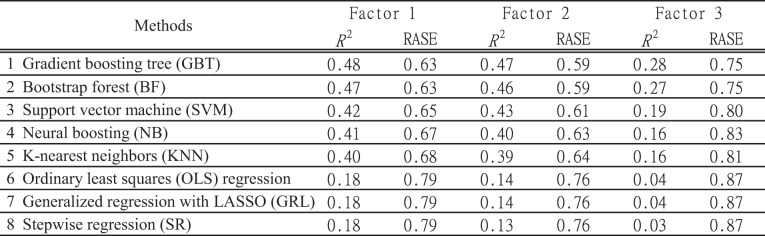
Comparison of regression models for predicting three factors of Texture, Sharpness, and Power, showing the coefficient of determination (*R*²) and root average squared error (RASE) for each method. Models are ranked by performance, with GBT achieving the highest predictive accuracy.

### Model validation

A GBT model was developed to model the relationship between acoustic features (input variables) and subjective impressions (output variables), leveraging GBT’s nonlinear learning^53^ and high-dimensional interaction analysis^54^. We evaluated the GBT with 10-fold cross-validation. The primary metric is the held-out R^2^ (fold mean ± Std. Dev.) based on out-of-fold predictions; RASE (fold mean ± Std. Dev.) is reported as a secondary metric. For overfitting diagnostics, we include the training R^2^ (in-fold; fold mean ± Std. Dev.) and the optimism gap (train − val; fold mean ± Std. Dev.). Hyperparameters were held fixed across folds, and all statistics are derived from held-out predictions. Full numerical results are provided in Table 8.

To assess performance above chance, we conducted a label-permutation test (multiple shuffles per outcome) using the identical pipeline. The permuted R^2^ distributions shown in Table 9 were centered near zero; the observed R^2^ in the cross validation (CV) exceeded the 95th percentile for all the factor components. This indicates learnable structure for all the factors.

**Table 8:**
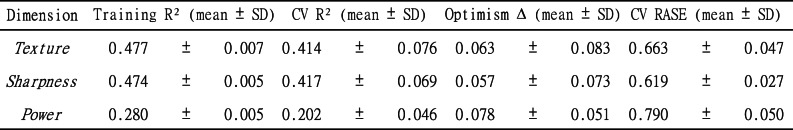
Cross-validated performance of the GBT based on 10-fold CV. Metrics are reported as held-out R^2^ (fold mean ± Std. Dev.) and RASE (fold mean ± Std. Dev.). For overfitting diagnostics we also report training R^2^ (fold mean ± Std. Dev.) and the optimism gap (train − val; fold mean ± Std. Dev.). Hyperparameters were held fixed across folds; all statistics are derived from held-out predictions.

**Table 9:**
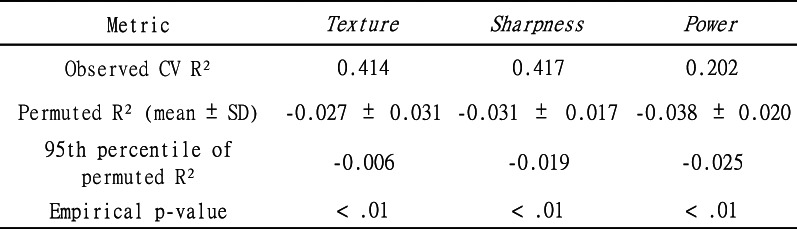
Permutation-based null distributions of CV R^2^. For each outcome, the labels were shuffled and the identical 10-fold CV pipeline was rerun 100 times. The table lists the mean, Std. Dev., 95th percentile, and the observed CV R^2^ from Table 7 with the corresponding p-value.

### Sensitivity analysis for subjective evaluation

**Fig. 7:**
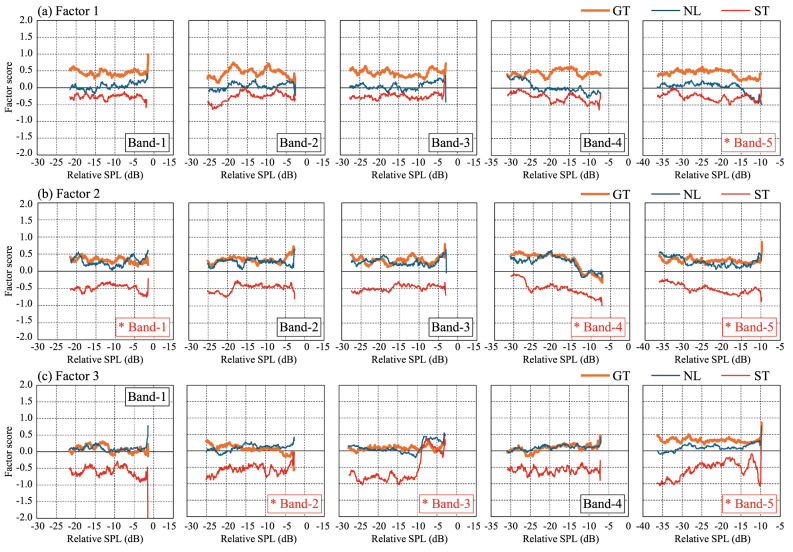
Sensitivity analysis results of the GBT model showing factor score responses to variations in band energy levels. Each subplot corresponds to a different frequency band (Band-1 (285 Hz) to Band-5 (4560 Hz)), where total energy across all frequency bands was normalized to 0 dB, and individual band energy levels are expressed as relative sound pressure levels. Parameters marked with red asterisks indicate those with a ratio of 0.1 or higher, as shown in Table 10.

**Fig. 8:**
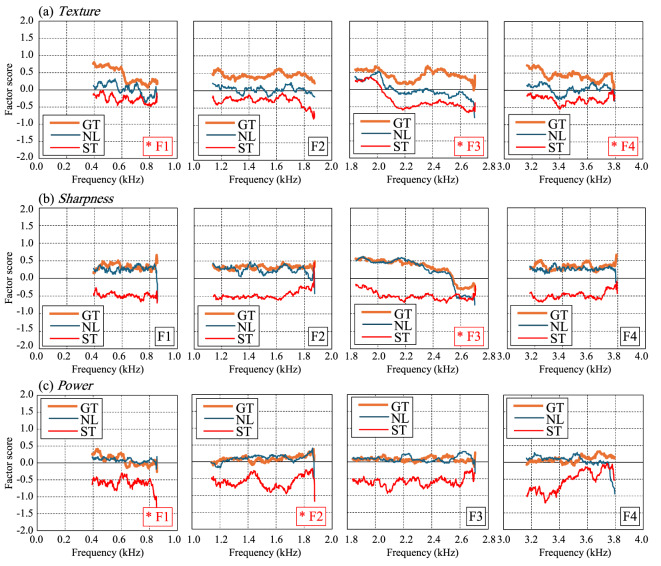
Sensitivity analysis results of the GBT model showing factor score responses to variations in formant frequencies. Each subplot corresponds to a different formant (F1 (500 HZ) to F4 (3000 HZ)), illustrating how changes in formant frequency influence subjective impressions. Parameters marked with red asterisks indicate those with a ratio of 0.1 or higher, as shown in Table 10.

Figure 7 shows the sensitivity curves generated by systematically varying input variables within empirically observed ranges, while holding others constant, while Fig. 8 presents outputs for formant frequencies. The response surfaces shown in Fig. 6 illustrate the predicted ratings across a 30 dB range of relative SPL values, derived from the natural dynamic range observed in recorded stimuli. In Fig. 7, total energy across all frequency bands was set to 0 dB, and each band’s energy was represented as a relative sound pressure level. In these figures, the conditions marked with red asterisks represent the parameters identified as important based on the GBT results. In this study, as shown in Table 10, importance was evaluated by the proportion of each parameter’s sum of squares relative to the total sum of squares. Parameters with a ratio of 0.1 or higher were considered for discussion below. Additionally, the order of the ratios presented in the table is also discussed in the following sections. Herein, a threshold of 0.1 was applied to the normalized Gain-based importance scores, consistent with common practices in XGBoost modeling (e.g., IBM Developer, 2023)^55^, to identify features with practically meaningful influence.

For Texture, in Band-5 (Fig. 7(a), 4560 Hz), GT and NL conditions showed a decline in scores when sound pressure exceeded − 15 dB, indicating a more positive texture impression. Regarding formant frequencies (Fig. 8(a)), higher F3 (2000 Hz) values in NL and ST led to a more positive impression, a trend also observed in F1 (500 Hz) and F4 (3000 Hz) for GT. This suggests that the formant order affecting texture perception varies by string type. Additionally, formant frequencies, particularly F1 (500 Hz), F3 (2000 Hz), and F4 (3000 Hz), exhibited a stronger influence on texture perception than band energy.

For Sharpness, in Bands 4 (2280 Hz) and 5 (4560 Hz), particularly in ST, higher sound pressure lowered the factor score, enhancing Sharpness. This trend was also noticeable in GT and NL for Band-4 (2280 Hz). In F3 (2000 Hz), ST showed increased Sharpness from ~ 2 kHz, whereas GT and NL exhibited this shift at ~ 2.4 kHz. Unlike Texture, only F3 (2000 Hz) showed a significant relationship, but both band energy and formant frequencies jointly influenced Sharpness perception.

For Power, in Bands 3 (1140 Hz) and 5 (4560 Hz), higher sound pressure, particularly in ST, increased the factor score, indicating greater Powerfulness. A similar trend was observed in GT and NL for Band-3. In terms of formant frequencies, higher F1 (500 Hz) values in GT and NL lowered Factor 3 scores, meaning lower F1 (500 Hz) corresponded to increased Power. Meanwhile, F2 (1000 Hz) in ST exhibited large fluctuations without a consistent trend. However, considering F3 (2000 Hz) and F4 (3000 Hz) in ST, higher formant frequencies generally increased Factor 3 scores, suggesting that higher mid-to-high formant frequencies enhance Power, whereas lower-order formants (~ 100 s Hz) reduce Power.

**Table 10:**
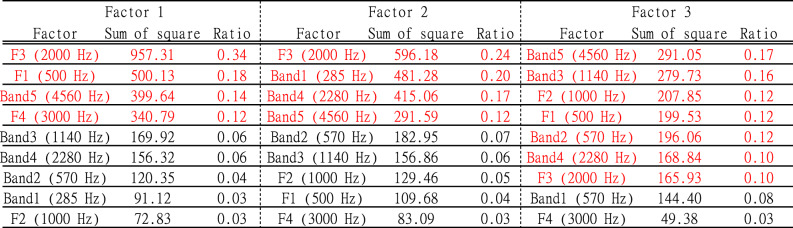
Summary of the sum of squares and their respective ratios for each parameter in the three factors of Texture, Sharpness and Power based on the GBT analysis. The table illustrates the contribution of each parameter to the total sum of squares, highlighting their relative importance in the model. Parameters shown in red indicate those with a ratio of 0.1 or higher.

## Discussion

The results of this study provide insights into the relationship between the acoustic characteristics of violin sounds and their subjective impressions. The findings reveal that the choice of string material significantly influences perceived texture, sharpness. Furthermore, regression and sensitivity analyses suggest that nonlinear modeling techniques, particularly GBT, are effective in capturing the complex relationships between acoustic and psychological variables.

### Influence of string and instrument type on subjective evaluation

The profile analysis demonstrated that steel strings tend to elicit brighter, clearer, and lighter textures compared to gut and nylon strings, which were perceived as coarser and more powerful. These results are consistent across multiple instruments, though the magnitude of differences varied. Instrument A, for instance, exhibited a pronounced distinction between gut/nylon and steel strings, whereas Instruments D and E showed minimal differences among string types. This suggests that while string material plays a crucial role in shaping timbral perception, the instrument’s inherent acoustic properties can either amplify or suppress these differences.

The factor analysis further supported these findings by identifying three distinct perceptual dimensions: Texture, Sharpness, and Power. The ANOVA results indicated that both instrument and string type significantly affected all three factors, with notable interactions. Texture and Sharpness were more predictable based on string type, while Power exhibited greater variability, suggesting that factors beyond basic acoustic energy and formant structure contribute to this perceptual dimension^56^.

These findings align with previous research indicating that formant frequency variations and spectral envelope characteristics play a critical role in timbre perception, particularly in distinguishing brightness and roughness^57^.

### Correlation between acoustic and subjective features

The analysis of Spearman’s correlation coefficients highlighted key relationships between formant frequencies, band energy, and subjective impressions. Texture was negatively correlated with formant frequencies, indicating that higher formant values were associated with smoother/finer Texture ratings. Texture was negatively associated with our mid-band envelope peaks. Within this framework, higher F1–F4 corresponded to smoother/finer Texture ratings. We do not claim prior evidence specifically on “texture”; rather, this pattern is consistent with work linking spectral-envelope shape/peak placement to timbral judgments (e.g., brightness, smoothness). To avoid over-interpretation, we report this as a model-based association within the observed range, not as a causal effect of “lower formant frequencies” per se. The present findings contribute new evidence to this area, aligning with prior research that highlights the role of spectral analysis and formant frequencies in texture perception^58^.

Similarly, Sharpness exhibited a moderate relationship with formant frequencies and higher band energy, particularly in the mid-to-high frequency range. This aligns with previous findings on formant tuning in singing, which suggest that spectral shape influences perceived timbre characteristics^59^. More generally, spectral-cue manipulations affect timbre judgements across species^60^. In contrast, Power showed weak and inconsistent associations with the static spectral descriptors considered here, suggesting contributions from temporal dynamics (e.g., attack, modulation) and instrument-specific resonances that were not explicitly modeled. We therefore report the observed nonlinear interaction patterns as model-level associations rather than attributing them to a specific mechanism.

While correlation analysis provided valuable insights, it was limited to linear relationships. The relatively low correlation values (~ 0.3–0.4) highlight the need for models capable of capturing nonlinear dependencies, prompting the use of regression techniques. The nonlinear nature of formant perception and spectral interactions has been established in previous research^58,61^, supporting the need for advanced predictive models to capture perceptual complexity in timbre.

### Effectiveness of nonlinear modeling and sensitivity analysis

Regression analysis confirmed that GBT outperformed other models, achieving the highest predictive power for Texture and Sharpness (*R*² ≈ 0.47) while yielding lower accuracy for Power. The advantage of GBT stems from its ability to model nonlinear interactions between multiple acoustic parameters, a capability that traditional regression methods such as Stepwise Regression and LASSO lack^59^. Computational models for musical sound analysis have demonstrated that linear approaches can be insufficient in capturing complex acoustic relationships^62^.

The sharp decline in performance observed with linear models (*R*² ≈ 0.1) suggests that the relationship between acoustic and subjective features is fundamentally nonlinear, consistent with previous research highlighting nonlinear dependencies in timbre modeling^63^.

The sensitivity analysis provided further insight into how specific acoustic features influence subjective impressions. Texture was most strongly affected by formant frequencies (F1 (500 HZ), F3 (2000 HZ), F4 (3000 HZ)) and high-frequency energy (Band-5), with greater sound pressure in these regions leading to more positive texture ratings, aligning with studies on the spectral cues underlying timbre discrimination^60^. Sharpness was predominantly influenced by Band-4 and Band-5, with higher energy levels, particularly in steel strings, enhancing perceived sharpness. Power was less predictable, though mid-to-high formant frequencies appeared to contribute positively to Dynamic perception, while low-frequency formants (~ F1 (500 HZ)) had the opposite effect, which aligns with research emphasizing the role of spectral characteristics, including formant structure and spectral envelope variations, in perceptual judgments of musical sounds^64^.

To clarify why string-type differences widen in the high-Sharpness regime, we discuss further as follows. Consistent with the paragraph above, increases in high-frequency band energy (Bands 4–5) produce a steeper effect for steel (ST)—that is, the factor score decreases more rapidly as level increases—and the F3-related rise in Sharpness starts earlier in ST (~ 2.0 kHz) than in GT/NL (~ 2.4 kHz). These two features—steeper high-band slopes and an earlier F3 onset in ST—account for the widening string-type differences as Sharpness increases. A practical interpretation is that steel tends to retain and transmit high-frequency content more efficiently, whereas gut/nylon dissipate it more, leading to larger Sharpness changes for the same incremental high-frequency increase in steel.

These results suggest that different perceptual dimensions of violin timbre are driven by distinct acoustic features. Texture perception relies heavily on spectral balance and formant frequencies, whereas Sharpness is more dependent on high-frequency energy, and Power is influenced not only by spectral characteristics but also by other factors such as temporal properties.

#### Limitations

The study demonstrates the importance of string selection in shaping violin timbre and highlights the role of instrument-specific acoustics in modulating these effects. From a methodological perspective, it underscores the necessity of nonlinear modeling techniques for accurately predicting subjective impressions from acoustic features.

However, the findings also reveal limitations in the current approach. While 40% of psychological variation was explained by acoustic features, the remaining 60% suggests contributions from additional factors such as musical experience, listener expectations, and cultural influences. Future studies should explore these aspects through neural or cognitive modeling and consider temporal dynamics, which were not explicitly analyzed in this study.

Regarding the recording device, the use of a directional microphone at relatively close proximity may have introduced low-frequency bias due to the proximity effect. While we standardized microphone placement to mitigate this, some spectral emphasis cannot be excluded.

Regarding the bowing-regime limitation, since bowing speed/force and contact point were fixed to improve comparability across 78 string–violin combinations, our models do not address conditions such as *pp*/*ff* dynamics, sul tasto/sul ponticello, vibrato, or onset transients. Given the known level- and excitation-dependent response of the bridge/body, the relative contributions of acoustic features to Texture/Sharpness/Power may change outside the controlled mf–mp regime. Future work should sample a sparse factorial grid of bowing parameters (e.g., 2 × 2 levels of speed/force with two contact points) or include performer-natural variations to test string × bowing interactions explicitly.

In addition, our acoustic-feature extraction adopted a measurement convention regarding envelope peaks and low-frequency resonances. Specifically, F1–F4 are treated as concise summaries of the mid-band spectral envelope derived with Praat/LPC—not as literal violin resonances^42,43^. Low-frequency body/air resonances—the air-cavity A0 (≈ 280–300 Hz) and body/plate modes around 400–800 Hz—are therefore quantified via band-energy measures (190–380 Hz; 380–760 Hz) rather than as explicit peak frequencies^40,41^. This separation improves robustness for sustained tones, but it also means that small shifts in A0/B1 ± frequencies are captured only indirectly, and that the mapping between F1–F4 and physical modes is instrument- and setting-dependent.

The same string sets were used sequentially across different instruments, which may have introduced minor effects due to material fatigue or subtle changes in string tension and elasticity over time. While we monitored string condition and replaced worn strings when necessary, full counterbalancing of the instrument–string combinations was not feasible. As such, we cannot fully exclude the possibility that these time-related effects contributed in part to the observed acoustic differences.

Furthermore, while the study successfully identified three perceptual dimensions—Texture, Sharpness, and Power—it did not examine how these dimensions relate to musical effectiveness or listener preference. These axes were derived purely from semantic evaluation, and no preference judgments were collected. As such, the findings should not be interpreted as implying musical desirability. Future work could explore how these perceptual constructs align with subjective notions of musical quality or expressiveness.

Finally, although the GBT model demonstrated strong predictive performance, its outputs are not inherently interpretable in terms of probabilistic inference or uncertainty. Bayesian regression approaches could offer improved transparency, robustness, and generalizability by incorporating prior knowledge and quantifying uncertainty. These represent promising directions for future model development.

Overall, this research provides a robust framework for understanding violin timbre perception, offering insights for instrument design, string selection, and computational timbre modeling. The strong predictive performance of GBT suggests that machine learning techniques will continue to play a crucial role in advancing acoustic-perceptual research.

## Data Availability

The datasets used and/or analyzed during the present study are available from the corresponding author upon reasonable request.
